# Severe anaemia after gastric biopsy in an infant with eosinophilic gastritis

**DOI:** 10.1186/s13052-019-0661-7

**Published:** 2019-06-06

**Authors:** Alessandro Daidone, Egidio Barbi, Vincenzo Villanacci, Grazia Di Leo

**Affiliations:** 1 University of Trieste, Trieste, Italy; 2 Institute for Maternal and Child Health - IRCCS “Burlo Garofolo”, Trieste, Italy; 3 Institute of pathology, Spedali Civili Brescia, Brescia, Italy

**Keywords:** Eosinophilic gastritis, Anaemia, Biopsy, Endoscopy, Emergency, Gastroenterology, Pediatric

## Abstract

**Background:**

Eosinophilic gastrointestinal disorders (EGID) are characterized by eosinophilic inflammation and are subclassified according to the affected site(s) as eosinophilic esophagitis, eosinophilic gastritis, eosinophilic enteritis and eosinophilic colitis. Clinical presentation includes dyspeptic symptoms, vomiting, abdominal pain, diarrhoea and gastrointestinal bleeding. Peripheral eosinophilia is usually found but is not required for the diagnosis. The treatment is based on dietary elimination therapy, consisting of removal of common food triggers, most frequently cow’s milk in infants. Corticosteroids are used as first line drug therapy in EG if dietary therapy fails to achieve an adequate clinical response or is impractical.

**Case presentation:**

A four month old infant was admitted for an episode of melena and hematemesis. An esophagogastroduodenoscopy showed haemorrhagic gastritis with ulcerative lesions and fibrin. A significant gastric bleeding was noted after the procedure. The gastric mucosa biopsies showed an eosinophilic infiltration.

**Conclusions:**

A clinically relevant anaemia is a quite rare complication in infants with eosinophilic gastritis and a biopsy may worsen bleeding, to a potentially severe level of low haemoglobin. In infants with low haemoglobin levels and suspect eosinophilic gastritis a watchful follow up after the biopsy should be considered, as well as the possibility of postponing the biopsy to reduce the bleeding risk.

## Background

Eosinophilic gastrointestinal disorders (EGID) are characterized pathologically by an eosinophilic infiltration in mucosal biopsies. EGID are subclassified according to the affected site(s) as eosinophilic esophagitis, eosinophilic gastritis (EG), eosinophoilic enteritis and eosinophilic colitis [1].

Clinical presentation includes dyspeptic symptoms, vomiting, abdominal pain, diarrhoea and gastrointestinal bleeding [2].

Peripheral eosinophilia is usually found but is not required for the diagnosis [3].

Treatment in infants is based on dietary elimination therapy, consisting of removal of common food triggers, mostly cow’s milk protein, and feeding consisting of amino acid based formula administration [4].

Corticosteroids are used as first line drug therapy in EG if dietary therapy fails to achieve an adequate clinical response or is impractical [5].

## Case presentation

A four month infant was admitted to the emergency department for an episode of melena.

Her past history was remarkable for recurrent post prandial vomiting, at about an hour’s distance from the meals, occasionally with minimal blood stain, started one month before, with reduced milk intake and a frank hematemesis the day before admission.

Her perinatal history was unremarkable. She was born after a normal pregnancy, at term, from cesarean section for breech presentation, weight at birth 2360 g. She was fed with infant cow milk based formula since the first weeks of life, growth rate was normal.

On admission she was well appearing with an unremarkable physical examination, the weight was 5930 g (25°-50°). A rhinoscopy ruled out upper airways bleeding.

Complete blood count showed mild leukocytosis with monocytosis (WBC 12780/mmc, N 5170/mmc, L 538/mmc, M 2210/mmc, E 10/mmc) with Hb 10.1 g/dl and platelets 407.000/mmc.

Lab tests showed ESR 22 mm/h, CPR 69.2 mg/L, electrolyte, liver, kidney function and coagulation tests were normal. An abdominal ultrasonography was normal.

An EGDS showed hemorrhagic gastritis with ulcerative lesions and fibrin with normal esophageal and duodenal mucosa. A gastric bleeding, significant but not justifying an endoscopic treatment, was noted immediately after the gastric biopsy.

Feeding was stopped for 24 h and an empirical proton pump inhibitor treatment started.

Six hours after the procedure, a complete blood count showed Hb 7.4 g/dl.

**Fig. 1:**
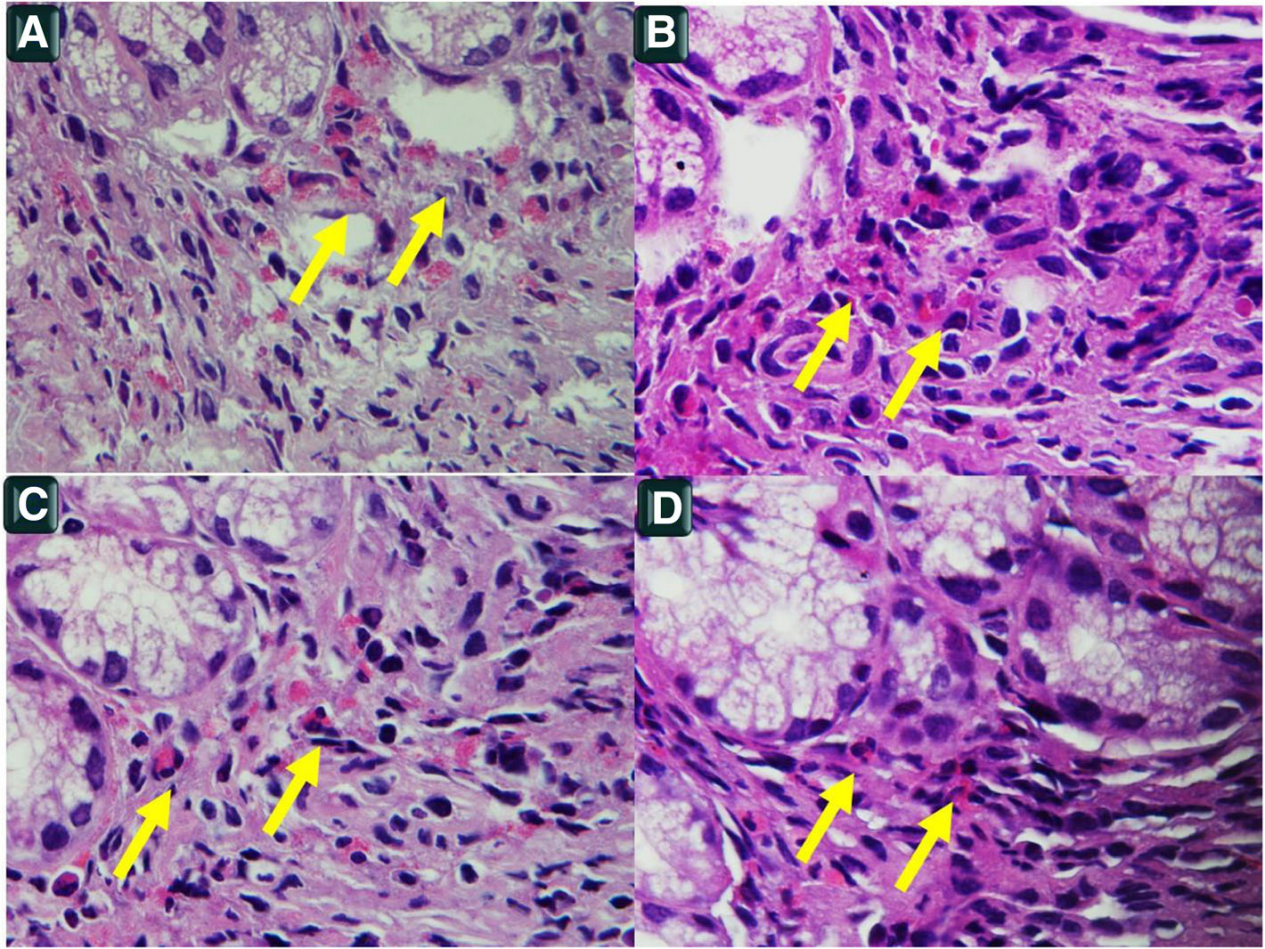
**a**-**b**-**c**-**d**: Pictures about the presence of eosinophils in the lamina propria of the stomach; in any figure the number of eosinophils (arrows) is over 5 for HPF at 100 x, H&E

The gastric mucosa biopsies showed an eosinophil infiltration compatible with diagnosis of EG (Fig. 1), esophageal and duodenal mucosa were normal.

## Discussion and conclusion

This report shows that severe anaemia, worsened after a biopsy, should be considered as a rare but potentially relevant complication in children with EG.

While bleeding or perforation are the most common adverse events after upper endoscopy with biopsy, this procedure is usually considered safe, with an overall low risk of complications. A clinically significant bleeding is estimated to be less than 0.5% in all cold mucosal biopsies [6].

This case suggests that children with EGID may present an increased mucosal fragility which favours bleeding, that may be related to a mucosal inflammatory pattern of lymphoid hyperplasia with eosinophil infiltration, facilitated by specific ligands such as 11 eotaxin-1 and C-X-C motif chemokine ligand 13 [7].

Hematemesis is usually not considered as typical symptom of EGID or among the most common causes of hematemesis.

While in a study of 28 patient with histological eosinophilic gastritis none presented hematemesis [4], other reports highlighted that hematemesis can be associated with EGID, in up to 70% of cases in a series of 13 Korean children, and with severe anaemia in two further case reports [8–10].

In our case the biopsy may have played a further role in causing severe anaemia suggesting that in children with low haemoglobin levels and suspect EG biopsies could be avoided or postponed to reduce the bleeding risk, and highlighting that whenever a biopsy is performed a watchful follow up should be considered.

The causes of gastrointestinal bleeding in an infant can be multiple [11], the most common being breastfeeding infant’s mother with cracked nipples (swallowed blood), erosive esophagitis due to gastroesophageal reflux disease, gastric or duodenal mucosal injury caused by infections or food allergy, malformations and portal cavernoma.

This case, along with recent literatures reports, reminds that EG should be considered among the possible causes. Gastrointestinal endoscopy is the most important diagnostic tool for the diagnosis of EGID due to its superior sensitivity and specificity [11]. Endoscopic abnormalities include nodular mucosa, erythema, and ulcers/erosions, but the mucosa may appear normal [1].

### Patient follow up

The baby was empirically treated with tranexamic acid and red blood cells were transfused. During 24 h fasting, she did not have new hematemesis and melena was less severe. The day after the procedure, oral feeding was started with an amino-acid based infant formula. Clinical condition improved with normalization of stools.

Three months later, while in milk-free diet the patient was in good clinical conditions. The EGDS was normal and the biopsy didn’t show the presence of eosinophils. At 14 months of age cow’s milk proteins were reintroduced without adverse events.

## Data Availability

All data generated or analysed during this study are included in this published article.

## Abbreviations

EG: Eosinophilic gastritis
EGDS: Esophagogastroduodenoscopy
EGID: Eosinophilic gastrointestinal disorders
